# Host Plant Selection by Larvae of the Muga Silk Moth, *Antheraea assamensis*, and the Role of the Antenna and Maxillary Palp

**DOI:** 10.1673/031.013.5201

**Published:** 2013-06-09

**Authors:** D. S. Bora, B. Deka, A. Sen

**Affiliations:** 1 Department of Life Sciences, Dibrugarh University, Dibrugarh-786004, Assam, India.; 2 National Chemical Laboratory, Pune, MH, India

**Keywords:** food choice, maxillae, Y-tube olfactometer

## Abstract

The importance of olfactory senses in food preference in fifth instar larvae of *Antheraea assamensis* Helfer (Lepidoptera: Saturniidae) was examined by subjecting larvae with only antennae or maxillary palpi after microsurgery to food and odor choice tests. Mean percent consumption, total consumption, and choice indices were used as parameters for drawing conclusions. The foods used were two hosts, two non-hosts, and a neutral medium (water). Both antennae and maxillary palpi were fully competent in preference for host plants, *Persea bombycina* Kostermans (Laurales: Lauraceae) and *Litsea polyantha* Juss, over the non-hosts, *Litsea grandifolia* Teschner and *Ziziphus jujuba* Miller (Rosales: Rhamnaceae). Both were competent in rejecting the non-hosts, *L. grandifolia* and *Z. jujuba*. The odor choice test was carried out using a Y-tube olfactometer and showed similar results to the ingestive tests. The results indicate the necessity of functional integration of a combination of olfactory and gustatory sensilla present in different peripheral organs in food acceptance by *A. assamensis* larvae.

## Introduction

During foraging, herbivorous insects perceive different information available in the natural environment, and a specialist might use specific biophysical and chemical cues provided by a host plant. Insects use various sensory systems to locate their hosts (Chapman 2003; Bullas-Appleton et al. 2004; Heisswolf et al. 2007; Jorgensen et al. 2007), and the degree of acceptability is probably based on the ability of the plants to elicit qualitatively different perceptions because of high chemosensory selectivity of output neurons (Dethier and Crnjar 1982; Reisenman et al. 2005). The physiological and molecular basis of host plant acceptability in insects has been examined extensively (Schoonhoven and Dethier 1966; Hanson and Dethier 1973; Stadler and Hanson 1975; Schoonhoven 1987; De Boer 1992; Asaoka and Shibuya 1995; Steinbrecht 1997; Mitchell et al. 1999; Ting et al. 2002; Shiraiwa 2008; Riffel et al. 2009). However, insects have a wide range of diet-breadth and, considering the phenomenon of chemosensory tuning to host-recognition cues (Glendinning et al. 2000; Del Campo and Miles 2003) and preferences for one specific compound present in all members of one plant family leading to oligophagy (Del Campo et al. 2001), every insect species may be considered to have its own key (Schoonhoven and Loon 2002). While it is well documented that the gustatory system plays a leading role in food discrimination (Hanson and Dethier 1973), insects need to rely on olfactory senses in natural habitats in order to reduce the cost of host plant searching. An insect approaches its host plant for feeding, oviposition, or shelter initially, most likely through perception of volatile chemicals. The antenna is the primary olfactory organ of insects, while the maxillary palp is a close range olfactory sensor in food selection, as has both olfactory and gustatory sensilla. In lepidopteran larvae, the antennae and maxillary palpi are known to contain about 80% of all the chemoreceptor cells (De Boer 2006).

Muga silkworm, *Antheraea assamensis* Helfer (Lepidoptera: Saturniidae), the producer of golden silk, is a lepidopteran insect endemic to northeastern India. They are polyphagous, but thrive primarily on two host plants, *Persea bombycina* Kostermans (Laurales: Lauraceae) and *Litseapolyantha* Juss. Restrictive feeding on a few plants might be the reason for its confinement to northeastern India only. Very few studies have been carried out so far regarding the feeding behavior of *A. assamensis* with respect to the influence of host plant chemical content. While Hazarika et al. (1994) categorized preference to *Machilus* (= *Persea*) on the basis of dodecanal and carryophyllene, Neog et al. (2011) showed a mixture of caryophyllene, decyl aldehyde, and dodecylaldehyde to be attractive for biting behavior of *A. assamensis* larvae. However, no work has been carried out to probe into the chemosensory basis of the restricted dietbreadth in *A. assamensis*. In this work, we report the role of the antenna and maxillary palp in food selection by *A. assamensis* larvae based on food and odor choice tests.

## Materials and Methods

### Insects

Larvae of *A. assamensis* that hatched from disease-free eggs obtained from the Government Sericulture Farm were cultured on leaves of its primary host plant, *P. bombycina*, grown in the botanical garden within the campus of the Department of Life Sciences, Dibrugarh University, Assam, India. Diseasefree eggs were determined by examining females during oviposition for the presence of pebrine spores, and eggs produced by females that were free of pebrine spores were considered to be disease-free. After the third moult, larvae were allowed to acclimatize to laboratory conditions (22–30° C) and the early fifth instar larvae were subjected to a food choice test using the method of De Boer and Hanson (1984) with necessary modifications and an odor choice test using a Y-tube olfactometer. The modifications were as follows: (1) in the test container, bamboo sticks were used for holding the fiber disks in place of a pin, and (2) the percent of larvae making a choice was calculated instead of choice index.

### Plants

For carrying out food choice, two host plants, *P. bombycina* and *L. polyantha*, and two nonhosts, *Litsea grandifolia* Teschner (Laurales: Lauraceae) and *Ziziphus jujuba* Miller (Rosales: Rhamnaceae), were selected.

### Plant extract preparation

Leaf extract was prepared by grinding 100 g of fresh leaves with 100 mL of distilled water in an electric grinder, and then filtering the mixture using double layered muslin cloth. Only freshly prepared filtrate was used in each bioassay.

### Ablation of the sensory organ

Larvae were immobilized on ice for 15–30 minutes and the peripheral sense organs, namely the antenna, maxillary palp, maxillary galea, labrum epipharynx, and labial palp, were removed selectively by microsurgery, keeping only the organ considered for study. Extirpations were performed on the two-dayold fifth instar larvae under a dissecting binocular microscope (Olympus, www.olympus.co.uk). After recovery, the larvae were allowed to feed normally on leaves of their primary host plant. Insects having unsuccessful operations were not consideredfurther. Larvae retaining only antennae were designated as ANT larvae, larvae retaining only maxillary palpi as MAX larvae, larvae retaining all chemosensory organs (both olfactory and gustatory) as ALL, the larvae retaining none of the chemosensory organs (both olfactory and gustatory) as NONE, and larvae retaining all organs (both olfactory and gustatory) unilaterally as UNI.

### Y-Tube olfactometer

**Figure 1.**
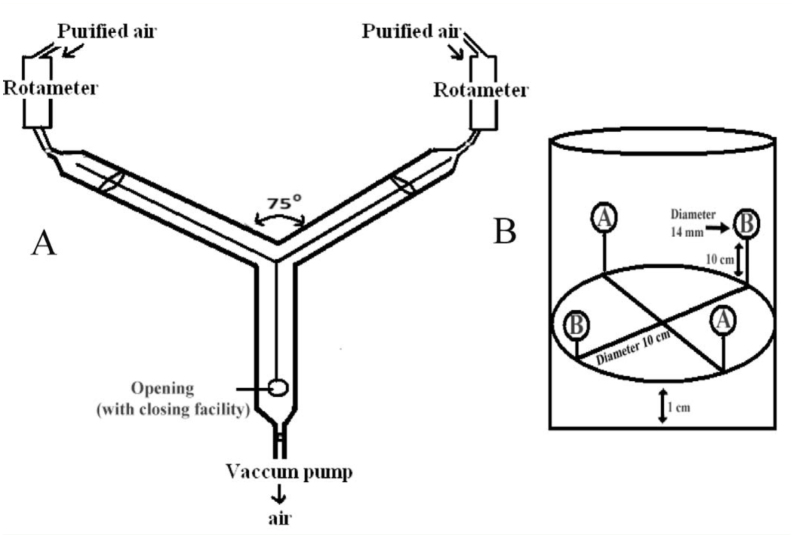
A: Scheme of Y-tube olfactometer. B: Scheme of leaf disk arrangement for food choice tests. High quality figures are available online.

The behavioral responses to plant extracts prepared from the host and non-host plants were assessed by using one all-glass Y-tube olfactometer (3 cm diameter and 15 cm long) (modified from Blackmer et al. 2004) (Figure 1A). Inside the tube, a glass wire of 0.3 cm thickness was attached in order to help the test insect to move towards the ends of the arms. One end of the stem of the Y-tube was connected to a vacuum pump (KNF, www.knf.com) to suck the air. The ends of the two arms of the Y-tube were connected to two adaptors made of Z-glass, and in each adaptor was placed a piece of Whatman filter paper (1 cm^2^). Each adaptor was connected to the Rotameter (Sigma-Aldrich, www.sigmaaldrich.com) in order to maintain a constant air-flow. The humidified air was first passed through charcoal filters and then through the rotameter into the adaptors attached to the arms of the Y-tube.

### Bioassays

**Food choice test.** Bioassay was done through a food choice test carried out in two ways. The two plants food choice test (dual choice test) was carried out between a host plant, *P. bombycina* or *L. polyantha* and a non-host, *L. grandifolia* or *Z. jujuba*. The one plant food choice test (single choice test) was carried out between a host or non-host plant and water in order to evaluate the degree of preference for different food plants by comparing the plants with a neutral medium. Water was used as the neutral medium, as it was used in the preparation of the leaf extract.

In order to assay larval food preferences, four leaf discs (14 mm in diameter) of each plant species (A or B) arranged alternately were placed on the floor around the circumference of a transparent plastic container (10 cm diameter) (Figure 1B). Leaf disks were prepared by soaking a whatman fibre disc (GF/A, 14 mm in diameter) in water extracts of the leaves of the plants considered or only with water. The leaf discs were fitted to the distal end of bamboo sticks, whose proximal ends were fixed on hard cardboard kept at 1 cm above the bottom of the container. The bamboo sticks were used to hold the leaf disc like a stem of a plant and to provide crawling space for the larvae. All the larvae were not fed for 2–4 hours before being subjected to the food choice test, and then were placed in the center of the floor of the container. When the larva had eaten about 50% of the area of one of the two plant species (A or B), the test was stopped. The amount of time it took, called T_50_, varied from 2 minutes to 1 hour from the start of the test. Tests were repeated with a minimum of 10 larvae. The 50% food consumption in T_50_ time was expressed in terms of percent consumption per minute using the unitary method of mathematical calculation. The percentage of choosing larvae was based on the number of larvae in one group opting for a particular food.

**Odor choice test.** In each assay testing orientation based on odor perception, a fifth instar larva belonging to any of the ALL, UNI, MAX, ANT, and NONE larval groups was released through a 2 cm opening located at the base of the stem of the Y-tube . Airflow was maintained in the Y-tube at 100 mL^-1^ from the opposite direction with the help of a rotameter and a vacuum pump. The freshly prepared extracts of leaves were applied to 1 × 1 cm pieces (Whatman No. 1) of filter paper. The filter paper disk containing the extract of the host plant was kept in one chamber (adapter), and the disk containing the extract of the nonhost plant was kept in the other chamber, so that the insect could perceive the odor moving along the airflow. The amount of time from the moment the insect started walking upwind until it reached 2 cm beyond the Y- junction of the Y-tube was recorded. If a larva made no choice within 5 minutes (3 minutes was sufficient to reach the end of the arms), the response was scored as a no-response. The experiments consisted of a minimum of 10 choices, and the insects showing no response were discarded. After each test, the entire setup was turned 180° to avoid any positional effects. Between each experiment, all parts of the set-up were washed and dried in an oven at 150° C. for 30 minutes.

### Statistical analysis

Preference based on mean percent consumption per minute was analyzed by one (*p* < 0.025) and two (*p* < 0.05) tailed MannWhitney tests. A chi-square test was performed between the percent of choosing larvae to show orientation preference. A nonparametric binomial test was used to determine significant differences between the numbers of larvae choosing different plants through odor perception. All analysis was done by SPSS 17.

## Results

### Food Choice Test

**Figure 2.**
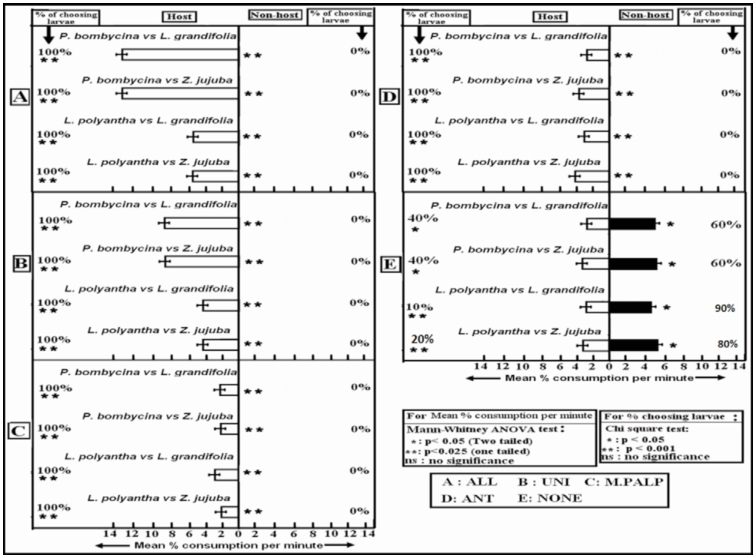
Mean percent consumption per minute and percentage of choosing larvae in the dual food choice test. High quality figures are available online.

***P. bombycina* vs. *L. grandifolia*.** Larvae were given a choice between host, *P. bombycina*, and non-host, *L. grandifolia*. ALL, UNI, ANT, and MAX larvae opted for *P. bombycina* (*p* < 0.025). The mean percent consumption per minute was significantly lower in ANT and MAX in comparison to that of UNI larvae (Figure 2C, D). The percent of larvae opting for *P. bombycina* was 100 in all larvae except NONE larvae, which failed to differentiate between *P. bombycina* and *L. grandifolia* (*p* < 0.05). 40% of NONE larvae opted for *P. bombycina*, while 60% opted for *L. grandifolia*. The mean percent consumption per minute was higher in the case of non-hosts (*p* < 0.05).

In the study for evaluating the preference towards the second primary host plant, *L. polyantha*, larvae were given a choice between *L .polyantha* and *L. grandifolia*. ALL, UNI, ANT, and MAX larvae opted for *L polyantha* (*p* < 0.025). Mean percent consumption per minute was reduced in MAX and ANT larvae (Figure 2C, D). NONE larvae failed to differentiate between the two choices (*p* < 0.05), and the percentages of larvae opting for both the plants were equal (50%) (Figure 2E). In NONE larvae, mean percent consumption per minute was higher in the case of non-hosts (*p* < 0.05).

***P. bombycina* or *L. polyantha* vs. *Z. jujuba*.** Larvae were given a choice between *P. bombycina* and *Z. jujuba*. ALL, UNI, ANT, and MAX larvae opted for *P. bombycina*, and the percent of larvae opting for *P. bombycina* was 100 in each case (*p* < 0.001) (Figure 2). Mean percent consumption of *P. bombycina* per minute by ANT and MAX larvae was reduced in comparison to that of UNI larvae (Figure 2BD). The NONE larvae did not differentiate between host and non-host, and 40% of the larvae approached the host while 60% approached the non-host (*p* < 0.05) (Figure 2E). In NONE larvae, the mean percent consumption per minute was higher in the case of nonhosts (*p* < 0.05).

When given a choice between *L. polyantha* and Z *jujuba*, ALL, UNI, ANT, and MAX larvae opted for *L. polyantha*, and the percent of larvae opting for *L. polyantha* was 100 in each case (*p* < 0.001). Mean food consumption per minute was minimum for MAX larvae (Figure 2C). NONE larvae did not differentiate between the host and non-host (Figure 2E). The percent of larvae opting for both the plants was equal, but the mean percent consumption per minute was higher in case of the non-host (*p* < 0.05).

***P. bombycina* or *L. polyantha* vs. water.** When larvae were given a choice between *P. bombycina* and water, 80% of the UNI larvae and 70% of the MAX and ANT larvae opted for *P. bombycina*. Only 20% of the NONE larvae opted for *P. bombycina*, and the remaining 80% opted for water (*p* < 0.001). The mean percent consumption per minute was higher in UNI and ALL larvae in the case of consumption of host plant against the consumption of water (*p* < 0.05). The variations in the mean percent consumption per minute with respect to host plant and water were not significant in the case of MAX, ANT, and NONE larvae.

**Figure 3.**
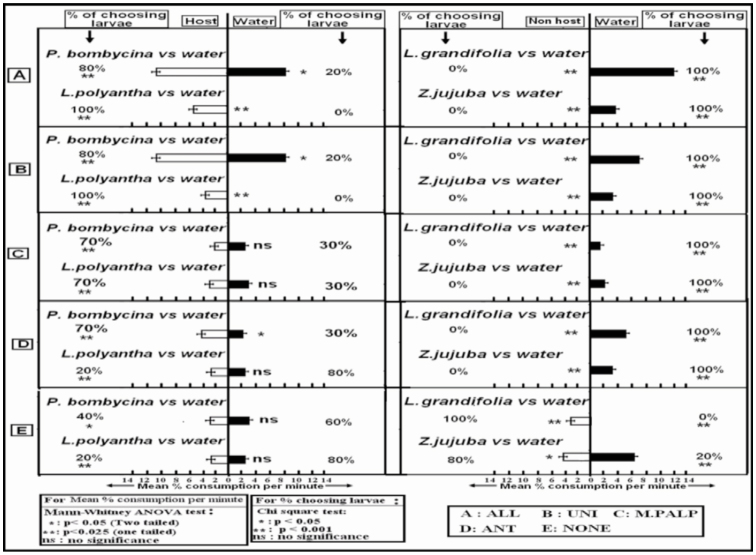
Mean percent consumption per minute and percentage of choosing larvae in the single plant food choice test. High quality figures are available online.

When larvae were given a choice between *L. polyantha* and water, ALL and UNI larvae opted for only *L. polyantha*, and the mean percent consumption per minute was highly significant (*p* < 0.025). 70% of MAX and 20% of ANT larvae opted for *L. polyantha*, and the rest opted for water. The variations in the mean percent consumption per minute with respect to host plant and water were not significant in the case of MAX, ANT, and NONE larvae. Similar to ANT larvae, only 20% of NONE larvae opted for *L. polyantha* (Figure 3).

***L. grandifolia* vs. water.** Larvae were given a choice between *L. grandifolia* and water. ALL, UNI, ANT, and MAX larvae opted for only water, and in each case none of the larvae opted for the non-host (*p* < 0.001). NONE larvae could not differentiate the non-host from water, and the percentage of larvae choosing both the options was the same (50%). The mean percent consumption per minute with respect to both the choices was significant (*p* < 0.05) (Figure 3).

***Z. jujuba* vs. water.** Larvae were given a choice between Z *jujuba* and water. ALL, UNI, ANT, and MAX larvae preferred only water over Z *jujuba* (*p* < 0.001), and mean percent consumption was highly significant (*p* < 0.025). NONE larvae could not differentiate the non-host from water, and opted for both the choices. The percent of larvae choosing the non-host was 40, and the percent choosing water was 60 (*p* < 0.05) (Figure 3). The variation in mean percent consumption between the two choices was significant (*p* < 0.05).

### Odor choice test

**Figure 4.**
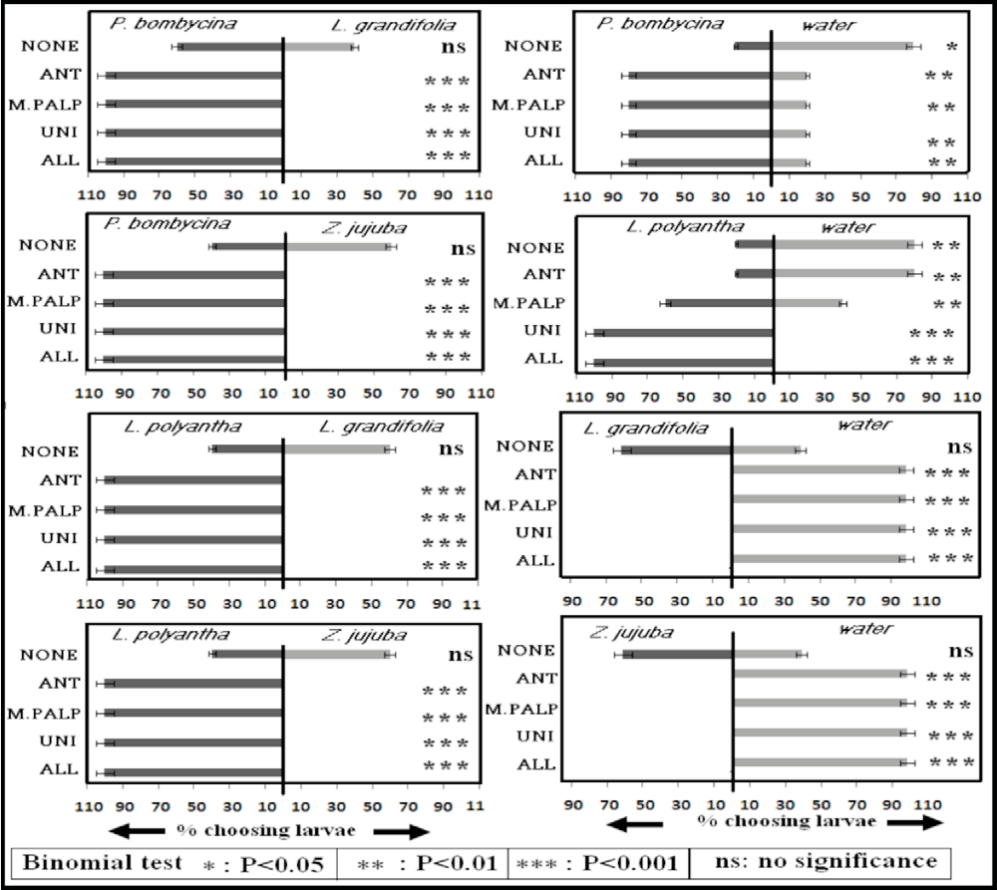
Percentage of larvae opting for odor choice in Y-tube olfactometer. High quality figures are available online.

When ablated larvae were given a choice between the odor of a host versus non-hosts, ALL, UNI, ANT, and MAX larvae opted for only the host plant odor (*p* < 0.001) and only NONE larvae opted for both the choices (*p* = 1.0). When the choice was given between the odor of a host plant versus water in the case of *P. bombycina*, 80% of ALL, UNI, ANT, and MAX larvae opted for the odor of *P. bombycina* (*p* < 0.01) and 80% of NONE larvae opted for water (*p* < 0.05). In the case of *L. polyantha*, 100% of ALL and UNI larvae (*p* < 0.001), 60% of MAX larvae (*p* < 0.05), and 20% of ANT (*p* < 0.05) and NONE larvae opted for *L. polyantha* (*p* < 0.05). In the case of the non-hosts, *L. grandifolia* and *Z. jujuba*, all the larvae opted for water except the NONE larvae, which opted for both the choices (*p* < 0.001) (Figure 4).

## Discussion

Larvae of *A. assamensis* feed on a very narrow range of host plants. Before the time of hatching, straw sticks containing the eggs of *A. assamensis* are tied to the tree trunk base. The newly hatched larvae, after taking several bites of the eggshells, crawl upwards in search of leaves, where they continue feeding until the leaf stock becomes exhausted. Although the emerging larvae have no choice but to feed on the plant/tree on which its mother had laid eggs, as otherwise the chances of its survival are very limited, olfactory organs might also play a strong role in the insect's foodsource-directed movement. The blend of volatile chemicals released by host plants either as constitutive or induced defense (Turling et al. 1995; Agarwal 1998; Walling 2000; Schoohoven et al. 2005) are perceived by olfactory organs. In order to understand the contribution of olfactory receptors in food preference behavior of *A. assamensis* larvae, ablation of all other chemosensory organs, leaving either the maxillary palp or antenna only, was done before subjecting the larvae to food and odor choice tests. As all the chemosensory organs are bilaterally represented, ablation of all chemosensory organs of one side should nullify the probable effect of surgery on food preference. Therefore, all tests of significance were carried out taking UNI larvae as the control group.

In the test related to the preference of a host plant over the non-host *L. grandifolia*, ALL, and UNI larvae showed preference for the host plant in the dual choice test. Therefore, each of the two peripheral organs were competent alone for mediating host preference, but none was absolutely necessary for the media-tion. Similarly, each of them were also competent in rejecting the non-host, as the NONE larvae failed to differentiate between host and non-host. In *Manduca sexta*, while antennae were recorded to mediate acceptance and rejection behavior for plants tested, maxillary palp mediated preference for normally rejected plant species (De Boer 2006). When preference for a host plant was studied by using one plant choice test, 70–100% of both ANT and MAX larvae preferred *P. bombycina* over water. In the absence of the antenna and maxillary palp, 80% of the NONE larvae preferred water over the host plants (Figure 3). This result confirmed that the larvae required either the antenna or the maxillary palp for mediating normal food preference. Many studies on the physiological basis of such oligophagy carried out in *M. sexta* larvae, a facultative specialist on solanaceous plants, have led to the conclusion that discrimination of food choice mostly depends on the chemical content of the food (De Boer et al. 1992), and a decision is made on a preconstruction recognition template, be it innate or acquired (Del Campo et al. 2001). The chemicals present in plants may also modulate functional significance of the chemosensory organs in particular and food choice behavior of the insect as a whole (Glendinning et al. 2000; Chapman 2003). For instance, when tuned to host-specialized chemical recognition cues, *M. sexta* remained oligophagous, but was otherwise polyphagous (Del Campo and Miles 2003).

When the choice was given between the host, *L. polyantha*, and water, only ablated larvae either with an antenna or a maxillary palp opted for both the choices. ALL and UNI larvae opted only for the host. While 70% of MAX larvae opted for both the hosts, *P. bombycina* and *L. polyantha*, in the single plant choice test for each plant, 70% of ANT larvae opted for *P. bombycina* in the choice test between *P. bombycina* and water. Only 20% ANT and NONE larvae opted for *L. polyantha* in the choice test between *L. polyantha* and water. This result might be due to the prior feeding history of the larvae. As all the larvae were grown on leaves of *P. bombycina* prior to the tests, antennae may have become tuned to the odor of *P. bombycina* only. Hence, when the larvae were exposed to the odor of *L. polyantha*, they could not recognize *L. polyantha* on first exposure as their host. Induction of feeding preference by diet was earlier reported in *M. sexta* (De Boer 1992). As per our studies using SEM, the maxillary palp of *A. assamensis* contains eight sensilla basiconica in a groove and, similar to other Lepidopterans (Dethier and Crnjar 1982), three out of them located centrally ,with a unique grooved structure are olfactory, and five located peripherally, each with a small terminal pore, are gustatory (unpublished data). The antennae of *A. assamensis* contain sensilla styloconica, sensilla basiconica, and basiconic pegs in the pedicel region (Hazarika and Bordoloi 1998). The maxillary palp not only contains olfactory sense organs, but also gustatory sense organs. Therefore, after the initial approach to the host plant based on olfactory perception, the taste receptors probably played a somewhat dominant role in the feedingacceptance decision-making process, and hence the MAX larvae exhibited equal preference towards both the host plants. Heisswolf et al. (2007) showed the stronger preference of a monophagous Chrysomelid beetle for its host plant to be based on contact cues rather than on odor cues.

Similar to *L. grandifolia*, in the food choice test for the non-host, *Z. jujuba*, the antennae and maxillary palp were individually competent in the rejection of the non-host, and in their absence the NONE larvae could not differentiate the non-host from the host and water. For the NONE larvae, the mean consumption per minute was higher during the consumption of the non-host and water disk. Thus, in the case of both the non-host plants, rejection behavior was regulated by the antennae and the maxillary palp. However, the mean consumption per minute in MAX and ANT larvae in many of the choice tests conducted were reduced in comparison to ALL and UNI larvae. This result might indicate that a complement of organs may be involved in food-acceptance decisions and biting and chewing activities (Boer and Hanson 1975) because only ALL and UNI larvae contained the full complement of the sensory organs involved in olfaction and gustation. The receptor neurons in other gustatory sensilla might contribute to lowering or enhancing the total excitatory input provided by odorant molecules. Biting activity is a temporal activity governed by the central nervous system. A central pattern generator for chewing located in the suboesophageal ganglion has been shown to be inhibited by thoracic input (Griss et al. 1991; Rowel and Simpson 1992; Rohrbacher 1994). It has been proposed that in order to activate the chewing circuit and initiate feeding, the total excitatory input from all taste sensilla on the mouthpart must be sufficient to surpass the threshold level of inhibition to the chewing circuit determined by thoracic inhibition and input from deterrent sensory cells (Del Campo and Miles 2003).

Like other insects (Saxena and Schoonhoven 1978, 1982; Asaoka and Akai 1991; Mondy et al. 1998; Asaoka 2000; Schoonhooven and Loon 2002; William III et al. 2010), odor cues emanated by the host plants may play an important role in host plant selection by *A. assamensis* larvae, owing to the participation of olfactory organs. In addition to the ingestive tests, when the ablated larvae were subjected to odor choice test using the Y-tube olfactometer, all larvae except the NONE larvae were attracted to only the host plant odor in dual plant choice tests. In conformity with the ingestive test, when the odor test was performed for host versus water, the percentage of MAX and ANT larvae opting for *P. bombycina* was equal to that of the control. However, the percentage of ANT and MAX larvae opting for *L. polyantha* was significantly reduced compared to that of the control. Except for in the case of *L. polyantha*, ANT larvae behaved similarly to NONE larvae. The results reconfirmed the tuning of the antennal receptor to host plant odor based on prior feeding history. Thus, in wild habitat, host plant chemicals play key roles in orientation and food selection through the participation of the antenna and maxillary palp in *A. assamensis*.

## Abbreviations:

ALL,: larvae retaining all chemosensory organs;
ANT,: larvae retaining only antennae;
MAX,: larvae retaining only maxillary palpi;
NONE,: larvae retaining none of the chemosensory organs;
UNI,: larvae retaining all organs unilaterally
